# Intracranial malignant melanoma: A report of 7 cases

**DOI:** 10.3892/ol.2015.3537

**Published:** 2015-07-24

**Authors:** YUNFENG MA, QIUPING GUI, SENYANG LANG

**Affiliations:** 1 Department of Neurology, Chinese PLA General Hospital, Beijing 100853, P.R. China; 2 Department of Pathology, Chinese PLA General Hospital, Beijing 100853, P.R. China; 3 Department of Psychology, Chinese PLA General Hospital, Beijing 100853, P.R. China

**Keywords:** malignant melanoma, CNS, CSF cytological pathology, imageology

## Abstract

The aim of the present study was to investigate the clinical diagnosis and treatment of intracranial malignant melanoma. For this purpose, the clinical manifestation, signs, cerebrospinal fluid (CSF) contents, imageology, pathological features, treatment and prognosis of 7 cases of intracranial malignant melanoma were analyzed in The Chinese PLA General Hospital (Beijing, China) from 1996 to 2013. All the melanoma cases were confirmed by histopathology, and CSF cytopathology demonstrated that there were 5 cases of primary malignant melanoma and 2 cases of secondary malignant melanoma. Among the patients, 4 presented with >1 pigmented nevus in the skin, and 1 presented with skin melanoma. Intracranial malignant melanoma mostly affects middle-aged males. CSF cytopathology and imageology (particularly enhanced magnetic resonance), are important tools in the diagnosis of the disease. Particularly, when a patient presents with a pigmented nevus in the skin and an abnormal lesion in the brain, a diagnosis of intracranial malignant melanoma should be considered.

## Introduction

Central nervous system (CNS) melanoma is a rare type of neoplasm. It is particularly rare in the brain compared with other areas of the CNS and accounts for 0.07–0.17% of all intracranial neoplasms. Depending on the behavior of the tumor, brain melanomas are classified into the following categories: i) Well-differentiated melanocytoma; ii) intermediate grade melanocytoma; and iii) primary malignant melanoma (1). CNS primary malignant melanoma only accounts for ~1% of all cases of melanoma (2). Primary CNS melanoma is generally diagnosed following the exclusion of a primary cutaneous or mucosal/retinal malignant melanoma, as differential histological diagnosis between primary and metastatic origins is often difficult (3). Primary leptomeningeal melanoma is an extremely rare type of primary intracranial melanoma, with a global incidence of 1 case per 20 million individuals (4). Primary leptomeningeal melanoma may be classified pathologically into two types based on the behaviour of the tumor; one type invades the pia mater diffusely and spreads into the subarachnoid space, while the other causes nodular tumors (5,6). Depending on the clinical presentation, treatment options include surgical resection, whole-brain radiation therapy, stereotactic radiosurgery and systemic therapy (7). Furthermore, the clinical outcome of patients with primary CNS melanoma is reported to be better than that of patients with metastatic disease owing to the possibility of long-term tumor control (8). Although the lifespan of patients with solid tumors may be prolonged with surgery following early diagnosis, generally, malignant melanomas present poor prognoses, partly due to their high rate of misdiagnosis. In the current study, 7 cases of histopathologically diagnosed intracranial malignant melanoma are presented.

## Case report

### 

#### Patients

The details of 7 patients with intracranial malignant melanoma were collected from January 1996 to March 2013 in The Chinese PLA General Hospital (Beijing, China). Among these patients, 3 were hospitalized in the department of neurology, and 4 in the department of neurosurgery. Primary and metastatic melanoma was the diagnosis for 5 and 2 of the patients, respectively. The cohort was formed by 1 female, who was affected with primary melanoma, and 6 males. Among the male patients, 4 were affected with primary melanoma, 1 with metastasis and 1 with post-surgical recurrence. The average age of onset of disease was 37.5 years. The lesions were identified in the following brain regions: The cerebellum (1 case), left frontal lobe (1 case), foramen magnum (1 case) and cerebral pia mater (3 cases). In 2 cases, the melanoma was accompanied by subarachnoid hemorrhage. Ethical approval was obtained from the Medical Ethics Community of the Chinese PLA General Hospital. In addition, written informed consent was obtained from all patients.

#### Clinical features

**Figure 1.**
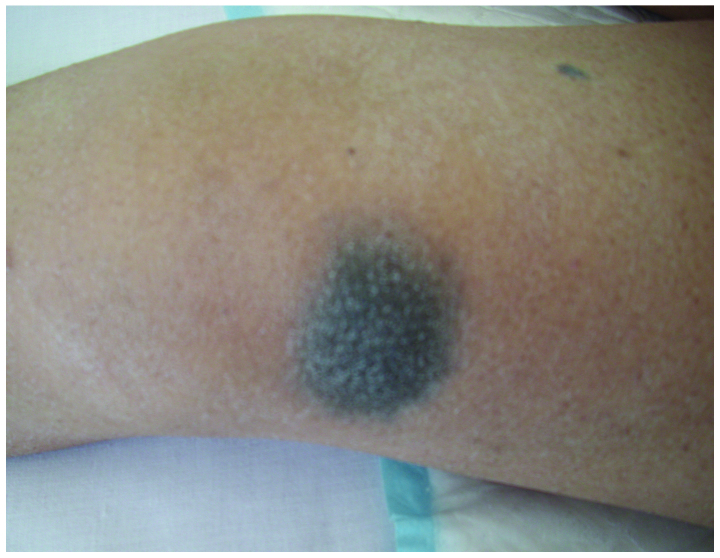
Case 6. Skin melanin pigmented nevus of 4×4 cm in size, observed in the left leg.

The majority of the cases presented an insidious onset, whilst 1 case had a sudden onset. The course of the disease was 2–3 months. The primary symptoms were headache (2 cases), upper digestive tract disturbances, including nausea and vomiting (2 cases), dizziness (2 cases) and neck and occipitalia pain (1 case). During the course of the disease, other symptoms, including nystagmus, diplopia, seizures and impaired vision and audition appeared in some but not all cases. In 4 of the 7 patients, >1 skin melanin pigmented nevus was observed (Fig. 1). The female patient had a history of epilepsy from childhood.

#### Cerebrospinal fluid (CSF) examination

In 3 cases, a solid tumor was not observed following a lumbar puncture. The intracranial pressure was markedly elevated, with all cases presenting intracranial pressure >300 mmH_2_O (normal range in adults, 100–180 mmH_2_O) (9), and 1 case >600 mmH_2_O. In the CSF, the following parameters were measured: Leukocyte numbers, 2–5×10^6^; glucose concentration, 0.17–2.44 mmol/l; levels of chlorides were normal; and protein concentration, 0.26–4.45 g/l. Cytological analysis of the CSF identified heterocysts with a large cellular volume and occasional double nuclei. Immunohistochemical analysis demonstrated positive staining for the melanoma marker human melanoma black 45 (HMB-45) and the leukocyte antigen cluster of differentiation 20 (CD20).

#### Imageology examination

**Figure 2.**
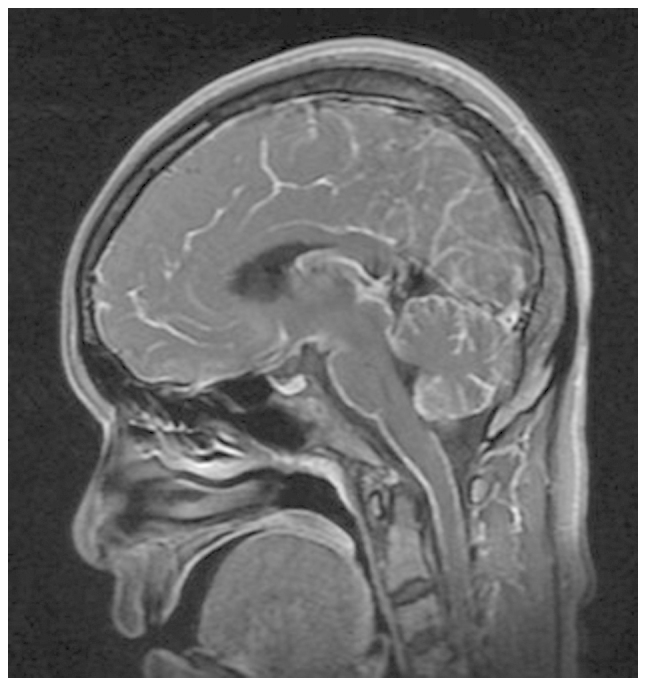
Case 6. Magnetic resonance imaging. Enhanced T1 sagittal view demonstrated enhanced diffused meninges.

Table I summarizes the results of the imageology performed on the 7 cases presented in the current report. Fig. 2 illustrates the enhanced diffused meninges of case 6, observed by magnetic resonance imaging (MRI) examination.

#### Pathology

**Figure 3.**
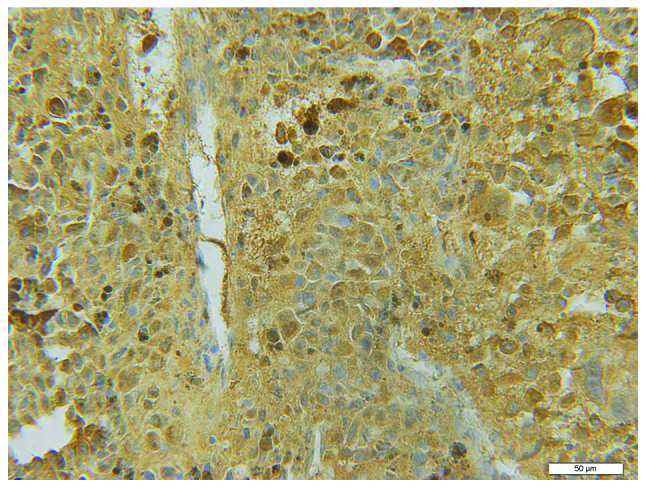
Case 3. Pathological examination of the left frontal lobe (magnification, ×40). Positive staining for S100 is observed.

**Figure 4.**
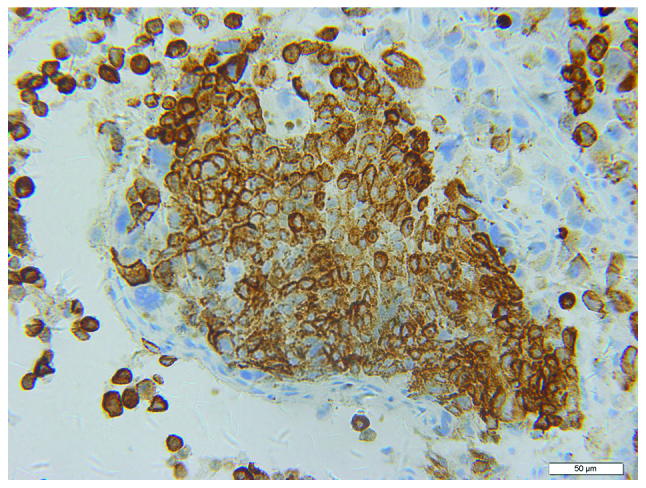
Case 3. Pathological analysis of the left frontal lobe demonstrates positive staining for human melanoma black 45 (magnification, ×40).

**Figure 5.**
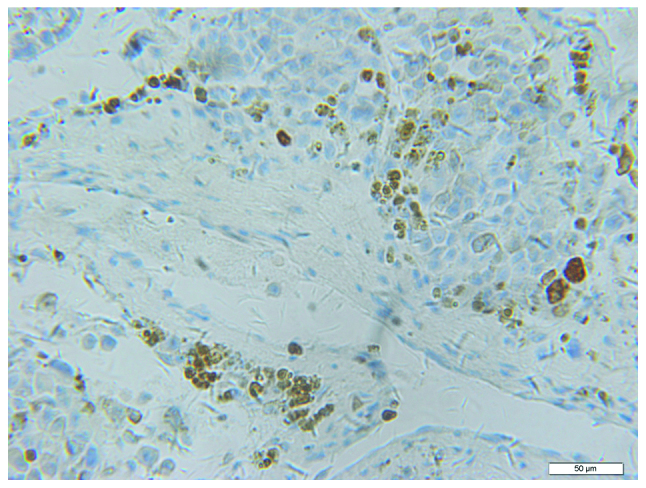
Case 3. Pathological examination of the left frontal lobe. Positive staining for Ki-67 is observed (magnification, ×40).

**Figure 6.**
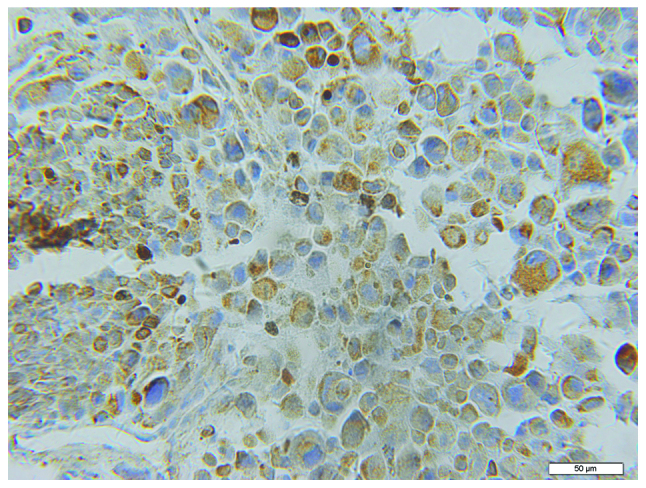
Case 3. Pathological analysis of the left frontal lobe demonstrates positive staining for vimentin (magnification, ×40).

During the surgery, the cerebral dura mater of the patients appeared to be black in 4 cases. In addition, the arachnoid appeared foliated and black in color in 4 cases. The tumors were observed to be tenacious and enveloped, rich in blood supply, and occasionally accompanied by obsolete hemorrhage. A diagnosis of melanoma was confirmed by postoperative pathology in 4 of the 7 patients, with 2 primary and 2 metastasized. CSF cytology confirmed the final diagnosis in the other 3 cases. Immunohistochemical analysis demonstrated positive staining for melan-A/S100 (Fig. 3); HMB-45 (Fig. 4); Ki-67 (50–75% positive staining) (Fig. 5); and vimentin (VIM) (Fig. 6). The staining for epithelial membrane antigen (EMA) was negative in 2 cases.

#### Treatment outcome

The symptoms of headache, dizziness, radicular pain and unsteady walking reduced in 4 cases following resection of the tumor mass. Since the treatment for leptomeninges melanoma was not effective, 1 patient discontinued the treatment, whereas 2 others refused chemoradiation, and were discharged following dehydration using mannitol.

#### Follow-up

Following surgery, 2 of the patients with metastatic malignant melanoma deceased after 2 months, while 2 survived for 9–15 months. Among the patients with leptomeninges melanoma, 1 patient succumbed several days following discharge, 1 survived for 4 months subsequent to chemotherapy and cytokine-induced killer cell therapy (four injections, every 2 days for 12 days), and 1 was unable to be contacted.

## Discussion

Melanoma originates from melanocytes, which are present in the human skin, mucous membranes, leptomeninges, cerebral parenchyma and uvea (2). Intracranial melanoma is classified as primary or metastatic, and primary CNS melanoma accounts for 1% of all melanomas (10). Primary melanoma derives from the leptomeningeal melanocytes, which are abundant in the skull base of the cervical cord (11,12). Primary meningeal melanoma are classified into 2 distinct groups, including diffuse tumors of the meninges, particularly around the base of the brain or spinal cord, which classically presents as communicating hydrocephalus; and discrete foci of melanoma, which are associated with diffuse meningeal involvement (13). Arantes *et al* (14) reported 13 cases of primary malignant melanoma derived from the pineal body. Pedersen *et al* (15) established the first model of melanoma driven by the oncogenic NRAS gene, which was expressed endogenously and did not require any additional genetic engineering of the mice, or exposure to carcinogens or tumor promoters. The authors reported 2 cases of children with melanoma of the CNS that presented mutations in the NRAS gene (15), and highlighted that melanoma of the CNS is associated with mutations in the GNAQ and GNA11 genes, whereas mutations in the NRAS gene are rare in adults (16,17).

Lang (18) reported that 90% of the cases of intracranial metastatic melanoma investigated were induced by skin melanoma metastases, whereas 2% arose from melanoma in the ocular region, and in 8%, the original lesion was not identified. In the current report, 4 patients presented with skin melanin pigmented nevus, 3 of which were affected by primary leptomeninges melanoma, and in 1 case the intracranial melanoma was secondary to the skin melanoma. Thus, in patients with skin melanin pigmented nevus concomitant with subarachnoid space hemorrhage, headache or intracranial hypertension, a diagnosis of intracranial malignant melanoma should be considered.

In the present study, the brain computed tomography (CT) plain scans of intracranial melanomas commonly displayed circular lesions with high densities, which were strengthened to varying degrees or presented a ring-shaped enhancement. In addition, the affected meninges displayed similar characteristics to those observed in subarachnoid space hemorrhage. The assessment of melanoma by MRI was complicated, since the melanin pigment is paramagnetic, and the results of the MRI mainly depend on the content of melanin pigment and the volume of blood in the tumor. Previous MRI results of metastatic melanomas were reviewed by Isiklar *et al* (19), who classified them into 4 groups as follows, based on the putative pattern: i) Melanotic pattern. Hyper, hypo and iso or hyperintense compared with cortex T1-, T2- and proton density-weighted images, respectively; ii) amelanotic pattern. Hypo or isointense compared with cortex on T1-weighted images, and hyper or isointense compared with cortex on T2-and proton density-weighted images; iii) indeterminate or mixed pattern. MRI characteristics do not conform to those of categories i) or ii); and iv) hematoma pattern. MRI features exhibit hematoma characteristics. The results of a brain MRI plain scan may be normal if solely the cerebral pia mater is involved (19). In these cases, enhancement MRI must be performed, since the meninges may be enhanced (20). In the present study, whole-body F-18 fluorodeoxyglucose (FDG) position emission tomography (PET)/CT eliminated the possibility of an extracranial origin for the melanoma lesions, and aided in the assessment of the therapeutic response in the early phase of the disease. Post-hoc PET/MR fusion images facilitated the correlation between the PET and MR images, and revealed the hypermetabolic lesions more accurately than the unenhanced PET/CT images did. Whole-body F-18 FDG PET/CT and post-hoc PET/MR images may aid clinicians in determining the best therapeutic strategy for patients with primary meningeal melanomatosis (21).

In the current report, 3 cases presented with primary leptomeninges melanoma, which is rare; its clinical manifestations, which are diverse and atypical, include the following: Subacute onset, behavior of chronic meningeal carcinomatosis, tendency to hemorrhage and repeated spontaneous subarachnoid space hemorrhage. Examination of the CSF demonstrated the following: Its color was pale yellow, due to repeated hemorrhage; sugar content was low; and protein concentration was >4.45 g/l (normal range, 0.15–0.45 g/l) (22). Further cytological examination of the CSF indicated the presence of heterocysts, and immunohistochemical analysis demonstrated positive staining for HMB-45, S100 and VIM. The latter results are important in the differential diagnosis of melanoma, particularly the positive staining for HMB-45, since this is a specific biomarker for melanoma (23).

The pathogenesis of leptomeninges melanoma is malignant, and its prognosis is poor (24). This is partly due to the lack of expertise in radio and chemotherapy in a large number of hospitals. However, it has not been reported that these treatments are capable of prolonging the lifespan of patients with leptomeninges melanoma. This disease should be differentiated from primary meninges melanocytoma, which is benign, and from neurocutaneous melanosis, which may cancerate to neurocutaneous melanoma. The patient of case 7 in the present study, who had a history of melanin pigmented nevus, presented with sheet melanin pigmented nevus on her face and body. The patient also suffered from epilepsy from a young age, which was revealed by right-sided rotation of the head, concomitant with lateral spasm. The brain CT scan of the patient demonstrated a high-density shadow in the left frontal lobe and sulcus. Although the presence of 5×10^9^ red cells/l in the CSF suggested a subarachnoid space hemorrhage, no alterations were observed in the 6 repeated brain CT scans. In addition, brain enhanced MRI suggested a focal enhanced lesion. Therefore, it was hypothesized that the patient suffered from meninges melanocytosis that cancerated to malignant, and developed a subarachnoid space hemorrhage. This hypothesis was supported by the fact that the high density of the brain CT scan corresponded to the presence of melanocytic neoplastic cells in the subarachnoid space. Furthermore, the cytological analysis of the CSF demonstrated the presence of heterocysts, which also suggested canceration.

Meningeal melanocytomas and primary CNS melanomas share a similar origin, and represent the benign and malignant ends of the spectrum, respectively (25). Malignant transformation of melanocytoma to malignant melanoma has been previously reported (26–29). Intracranial malignant melanoma often progresses, as it is not eradicated easily and tends to recur. Primary intracranial melanoma should be treated by thorough resection, followed by post-surgical radio and chemotherapy (30). Currently, the most common and effective chemotherapeutic agent is dacarbazine, which presents an effectiveness rate of 16–20%, and is administered intravenously following surgery or radiotherapy (31). Previous studies have demonstrated that stereotaxic radiosurgery (SRS) is able to treat 1–3 lesions, and is more effective at treating metastatic intracranial melanoma than traditional whole-brain radiotherapy, since a single exposure to a large dose of SRS may overcome resistance to radiation, limit the damage to peripheral brain tissue, and enable the control of focal transfer. SRS, on its own or with the addition of whole-brain radiotherapy, may prolong the lifespan of patients with metastatic intracranial melanoma, and improve the control rate of CNS lesions (31,32).

Salpietro *et al* (33) reported that specific immunotherapy was an important adjuvant method in the treatment of small residual malignant melanoma lesions, and presented low toxicity. High doses of IFNβ or IFNα-2b may improve disease control and prolong survival time, but the dosage required is disputed and difficult to tolerate. In 2008, Hunder *et al* (34) reported the bioremediation experienced by a patient with multiple metastasis melanoma, who was in remission for 2 years, but did not present with any metastatic lesion in the brain.

In conclusion, the prognosis of intracranial malignant melanoma is determined by the following factors: i) The type of lesion; ii) the involvement of the leptomeninges; iii) the extent of tumor excised; and iv) the molecular immunology borstel number 1 (MIB 1) antibody index, which is the most relevant factor for prognosis in this type of cancer (1).

## Figures and Tables

**Table I.** Imageology summary of 7 cases of intracranial malignant melanoma.

Case	CT	Enhanced CT	MRI	Enhanced MRI	MRA	DSA	PET-CT
1	Left cerebellum lateral high density	Uniformly enhanced	NA	NA	NA	NA	NA
2	Multiple metastatic tumor	NA	NA	NA	NA	NA	NA
3	Forehead lesion (with hemorrhage)	NA	Equal T1 short T2 uneven sign surrounded by forehead	NA	-	NA	NA
4	NA	-	Foramen magnum and right CPA lesion partially equal T1, short T2	NA	NA	-	NA
5	Left temporal hemorrhage with SAH	NA	Left temporal multiple short T1, short T2	NA	-	NA	NA
6	Left forehead sulcus high density	NA	-	Leptomeninges enhanced	-	-	-
7	SAH	Uniformly enhanced	-	Leptomeninges enhanced	NA	-	NA

Normal observations are indicated by the symbol -; NA, not assessed; CT, computed tomography; MRI, magnetic resonance imaging; MRA, magnetic radiology angiography; DSA, digital subtraction angiography; PET; position emission tomography; SAH, subarachnoid space hemorrhage; CPA, cerebellopontine angle.
